# A length-adjustable vacuum-powered artificial muscle for wearable physiotherapy assistance in infants

**DOI:** 10.3389/frobt.2023.1190387

**Published:** 2023-05-04

**Authors:** Samuel Dutra Gollob, Mijaíl Jaén Mendoza, Bon Ho Brandon Koo, Esteban Centeno, Emir A. Vela, Ellen T. Roche

**Affiliations:** ^1^ Department of Mechanical Engineering, Massachusetts Institute of Technology, Cambridge, MA, United States; ^2^ Department of Mechanical Engineering, Universidad de Ingenieria y Tecnologia, Lima, Peru; ^3^ Research Center in Bioengineering, Universidad de Ingenieria y Tecnologia, Lima, Peru; ^4^ Institute for Medical Engineering and Science, Massachusetts Institute of Technology, Cambridge, MA, United States

**Keywords:** soft robotics, artificial muscle, adaptable, growing, wearable

## Abstract

Soft pneumatic artificial muscles are increasingly popular in the field of soft robotics due to their light-weight, complex motions, and safe interfacing with humans. In this paper, we present a Vacuum-Powered Artificial Muscle (VPAM) with an adjustable operating length that offers adaptability throughout its use, particularly in settings with variable workspaces. To achieve the adjustable operating length, we designed the VPAM with a modular structure consisting of cells that can be clipped in a collapsed state and unclipped as desired. We then conducted a case study in infant physical therapy to demonstrate the capabilities of our actuator. We developed a dynamic model of the device and a model-informed open-loop control system, and validated their accuracy in a simulated patient setup. Our results showed that the VPAM maintains its performance as it grows. This is crucial in applications such as infant physical therapy where the device must adapt to the growth of the patient during a 6-month treatment regime without actuator replacement. The ability to adjust the length of the VPAM on demand offers a significant advantage over traditional fixed-length actuators, making it a promising solution for soft robotics. This actuator has potential for various applications that can leverage on demand expansion and shrinking, including exoskeletons, wearable devices, medical robots, and exploration robots.

## 1 Introduction

Exoskeletons and wearable devices for physiotherapeutic applications of many varieties are well described in literature, though most currently available exoskeletons for lower-limb physical therapy consist of rigid materials and rigid actuation schemes such as DC motors, gear boxes (Sancho-Pérez et al., 2016; Wu et al., 2016; Narayan and Kumar Dwivedy, 2021), cable transmission mechanisms (Asbeck et al., 2015; Ding et al., 2018; Di Natali et al., 2020; Wang et al., 2022), and bar mechanisms (Chaparro-Rico et al., 2015). These types of exoskeletons and mechanisms provide high forces, repeatable assistance and good structural support, but have drawbacks in patient comfort and safety due to heavy, rigid components that pose risks for pinching and other forms of mechanical injury. To overcome these risks, exoskeletons with fully soft structures have been described (Polygerinos et al., 2015; Sridar et al., 2017; Nguyen and Zhang, 2020).

Within the sub-field of wearable devices for physical rehabilitation of infants, soft or conformable materials present an alternative to rigid exoskeletons (Goldfield et al., 2012). Such devices can be passive (Sargent et al., 2015; Lobo et al., 2016;), which limits their use to supports or resistance bands, while others use soft actuation (Goldfield et al., 2012; Park et al., 2014; Subramanyam et al., 2015; Kokkoni et al., 2020). The use of soft robotic systems for physiotherapy exoskeletons in infants is desirable, as they reduce the risk of injury, are lightweight and inexpensive, and conform well to the body of the wearer. Despite the variety of soft platforms for infant rehabilitation, none address the key issue of rapid patient growth, requiring new devices to be designed as patients grow. This work attempts to address this key limitation in soft exoskeletons for infant physiotherapy by presenting a length-adjustable soft robotic actuator that easily adjusts to grow with patients.

The key components of a soft robotic exoskeleton are soft or flexible actuators, also called artificial muscles (AMs). There is a variety of AMs in the literature, with one of the most popular forms being Pneumatic Artificial Muscles (PAMs), which consist of a bladder that is pressurized pneumatically, leading to various types of motion. The most common motion produced by an artificial muscle is contraction with ratios of 35% and 60% for elastomeric soft actuators and membrane-based soft actuators respectively (Chou and Hannaford, 1996; Niiyama et al., 2014; Rus and Tolley, 2015; Hawkes et al., 2016; Drotman et al., 2019; Yang et al., 2019; Zhu et al., 2019). Other examples of AMs produce bending motion using asymmetric configurations (Galloway et al., 2013; Mosadegh et al., 2014; Khin et al., 2017; Sridar et al., 2017) and twisting motion through helical fiber reinforcement (Galloway et al., 2013; Khin et al., 2017).

In comparison to PAMs that are actuated with positive pressure, Vacuum Pneumatic Artificial Muscles (VPAMs) use negative pressure. Some attractive features of VPAMs are the ability to work in space-limited scenarios, high force to weight ratio, and high speed. VPAMs are also safer than positive pressure PAMs as wearable actuators, as their negative pressure means they do not explode upon failure. VPAMs perform a variety of motions such as bending (Li et al., 2017; Usevitch et al., 2018) and twisting (Jiao et al., 2019; Jiao et al., 2021). Linear contraction can be generated using design features including buckling elastomeric structures (Yang et al., 2016; Yang et al., 2017), foldable structures (Zhang et al., 2021), equidistant rings (Felt et al., 2018; Lee and Rodrigue, 2018; Usevitch et al., 2018; Li et al., 2019), internal origami skeleton (Li et al., 2017; Mendoza et al., 2021), springs (Kulasekera et al., 2020; Kulasekera et al., 2021) and deployable structures (Yu et al., 2021).

In general, there are two main configurations in VPAMs: Bellows Vacuum actuators (BVAs) and Fluid-driven Origami Artificial Muscles (FOAMs). BVAs offer high forces (≈70 N) and high contraction ratios (≈90%) using an inextensible but flexible skin with a tubular shape with internal rings spaced evenly (Felt et al., 2018; Lee and Rodrigue, 2018). Their performance is hindered as compression of the membrane is unpredictable, and leads to non-uniform contraction and high hysteresis. FOAMs offer large contraction ratios up to 90% by encapsulating a zigzag skeleton in a skin (Li et al., 2017). Replacing the skeleton for a spring or a deployable structure leads to contraction ratios under 40% and 80%, respectively (Kulasekera et al., 2020; Yu et al., 2021). Our group previously reported a low-profile VPAM based on BVAs and FOAMs that was designed for rehabilitation of infants less than 3 months of age and produced a maximum force of 26 N (Mendoza et al., 2021).

Although significant progress has been achieved in AMs, there remains a scientific challenge to develop AMs capable of adapting to a patient’s body in a wearable setting, particularly in growing infants. For our particular technological design challenge, we chose to focus on infants with motor paralysis in the lower limbs, in conditions such as cerebral palsy and myelomeningocele. For lower-limb paralysis in infants, a key form of physiotherapy is Range of Motion (ROM) exercises, which consist of the active bending of the joints in their full range of motion (usually by a caretaker or health professional) to ensure a preservation of joint mobility as the child’s limbs develop (Seattle Children’s, 2018). In the case of myelomeningocele, infants suffer from paralysis, and for the first 6 months of life ROM exercises are the primary form of physiotherapy, after which other forms of physiotherapy are introduced (Tappit-Emas, 2008). In this work, we focus on the ROM exercise of the knee joint, which is performed in the prone position and requires a large range of motion, as well as a smooth motion trajectory for patient safety (Seattle Children’s, 2018). With these parameters in mind, we chose the BVA due to its high contraction ratio as a VPAM. In comparison to rigid mechanisms and conventional PAMs, the BVA offers a variety of safety benefits, as it cannot exceed its maximum contraction, remains equally deformable throughout its actuation states, and as previously mentioned, does not suffer the explosion risk that positive-pressure actuators do. On top of greater safety, the light and soft materials used, allow for a portable assembly that is more comfortable for the wearer than the existing rigid approaches. Finally, the modularized form factor of the BVA lends itself to our adjustable length design, as will be explained in later sections.

In this work, we report a single BVA capable of adjustable functional lengths and present a case study of its application for ROM knee joint exercises for 0-to-6 month-old infants. We first describe the design in further detail and characterize the actuator at various length states. Additionally, we present a dynamic model and an open-loop control approach developed to predict a suitable actuator pressure input to achieve a target knee motion. Finally, we validate the model through a set of experiments on a model leg. We find that the clipping BVA functionality allows the same actuator to be used on a growing simulated patient, and that our model is able to predict the desired leg motion in an open-loop fashion, accounting for the change in BVA length.

## 2 Materials and methods

### 2.1 Working principle and design requirements

**FIGURE 1:**
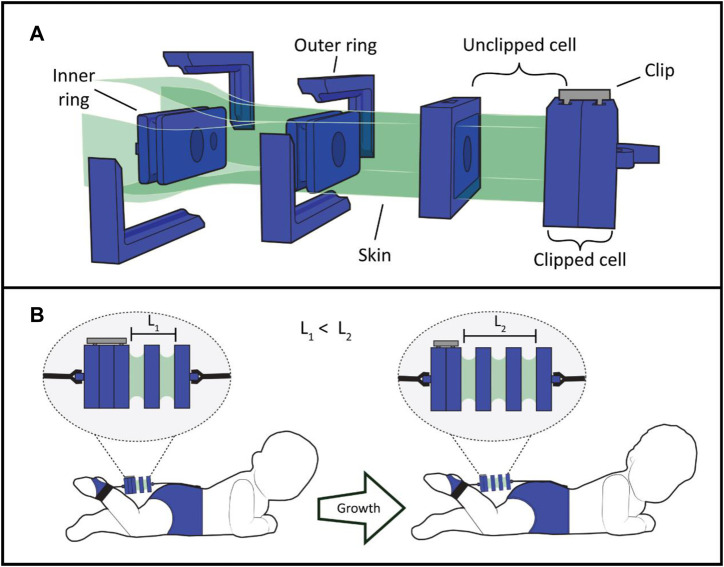
Working principle for the BVA presented in this work, including **(A)** Assembly view of the actuator with labelled components, and **(B)** A concept drawing of how the same actuator can be used on a growing patient by unclipping cells over time. A fabric sleeve would be placed around the actuator (See Supplementary Material).

The BVA presented here is a low-profile soft artificial muscle composed by evenly spaced cells with a rectangular cross-sectional area. Our BVA consists of five components: a flexible membrane or skin, internal rings, external rings, and anchoring clips (Figure 1A). Standard BVA designs often include solely internal rings, evenly-spaced to create a chain of contractile cells which can be tuned based on cell spacing (Felt et al., 2018; Gollob et al., 2021). The key unique features of our design are its functional external rings and clips, which allow this design to be length-adjustable, by clipping or unclipping actuator cells (Figure 1B). The internal rings still provide necessary support for the skin, but the external rings envelop the internal rings from outside the skin (See Supplementary Material for fabrication details). The clipping concept works by sliding the clips in grooves placed on the external rings, thus attaching two neighboring rings and in essence deactivating the clipped cell. More cells can be clipped at once with a clip that spans the length of multiple rings.

The clipping mechanism allows a BVA to adjust its contractile length. A BVA can have clipped cells to work in a scenario with limited space and unclip cells to lengthen and produce a higher absolute contraction. This feature can offer an advantage over fixed-length soft robots in terms of reusability. In our case study, we demonstrate this with an active length range of 30 mm–40 mm (2–3 unclipped cells) on a growing patient model (Figure 1B; Supplementary Video S1), though this concept can be extended to BVAs of arbitrary length and cross-sectional area.

For our case study of infants with myelomeningocele from 0 to 6 months of age, we worked with clinicians to define the requirements for the knee exercise such as the time for leg flexion-relaxation, the ROM, and a desired trajectory (see Supplementary Table S4; Supplementary Material). A key detail, which led us to select a sinusoidal trajectory was the desire for smooth and continuous motion, without sharp acceleration. Considering this information, we designed the BVA with a rectangular cross-sectional area for its low profile and greater rotational stability. The required actuator length for each month was calculated based on biometric data on infant limb dimensions (Sun and Jensen, 1994), as well as a theoretical range of motion and output force magnitude requirements, informed by an existing generalized model of bellows actuators (Gollob et al., 2021).

As for the design of the interface with the patient, we consulted with our clinical collaborators again to define the anchoring locations for the actuator across the knee joint, anchoring near the end of the calf and the base of the thigh, near hips (see Supplementary Table S4; Supplementary Material). This was also informed by the lever arm concept and our quasi-static model, would allow the contractile actuator to produce low forces (<8 N) for the exercise (see Supplementary Section S1.4), increasing overall assembly safety. The Supplementary Video S1 shows a mock-up version of this design for visualization purposes. We note that despite this lower force, there may still be loads off of the flexion-extension axis of the knee joint, which would present a risk of injury for the patient and would be addressed in future versions of the design, likely through more robust anchor design and support added to the knee joint to prevent off-axis bending.

### 2.2 Model-based open-loop control of actuator

#### 2.2.1 Dynamic model of the leg

As a prelude to the Back-Solver model in the next section, a dynamic leg model was developed. It represents the lower leg bending at the knee and is composed of a pinned rotating mass and a rotational damping element to as a lumped loss factor. A contractile actuator is attached across the knee joint, with anchoring locations along where the calf and thigh would be, as explained above. The actuator outputs a nonlinear tensile force calculated from its current length and pressure, based on an empirically-derived function, and that force is converted to a torque trigonometrically. After formulation, the dynamic model was validated through dynamic experiments to ensure it accurately represented the system for our purposes (see Supplementary Section S1.6).

The greatest challenge for model accuracy is the proper characterization of the actuator’s output force, as it is known from previous work that force is non-linear with regards to actuator contraction, while being linearly proportional to pressure (Felt et al., 2018; Gollob et al., 2021). This nonlinear relationship between output force and actuator displacement is referred to as the Force-Contraction Profile (FCP). Though previous work has also demonstrated a method for predicting the nonlinear FCP of an actuator from its geometry (Gollob et al., 2021), our application proved incompatible with actuator modelling given the variability in actuator construction, small inaccuracies in actuator measurements, and the overall sensitivity of the system to errors in force output (given its open-loop operation). Rather, the output force-contraction for the actuator was derived empirically.

**FIGURE 2:**
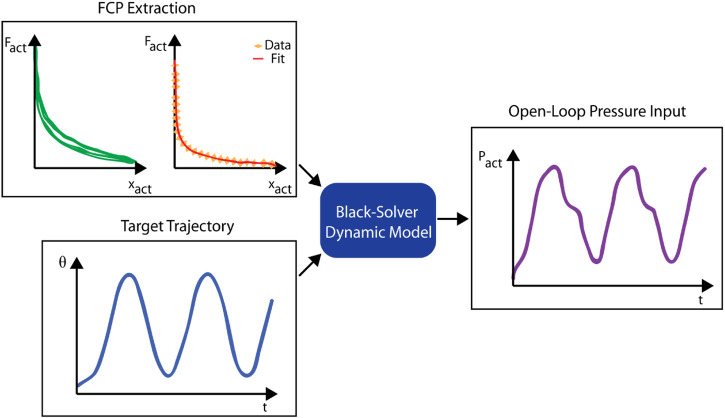
Schematic of the workflow for running the Back-Solver model, including the FCP (the hysteretic FCP shape and the final fitted FCP using the lower part of the hysteretic curve), and the target trajectory as the input formats of the model, as well as the open-loop pressure input as the output formats of the model. $F_{act}$ , $x_{act}$ , and $P_{act}$ stand for actuator force, contraction, and pressure respectively.

This process, further detailed in the Experimental Methods Section (2.3.4) and depicted in Figure 2, involves extracting an implicit actuator output force curve given the measured leg motion in an experimental setup of the knee-flexion exercise. This empirical approach to FCP derivation has a few benefits for on-site use in a clinical setting over a model-based or other direct measurement approaches. Firstly, it requires no additional equipment from the user other than the sensors that would already be present in the system (IMU and pressure sensor), unlike a force-based method that would require a mechanical testing setup. Secondly, this method is specific to the assembly, and so can absorb some of the discrepancy between the model’s assumptions and the reality about system parameters. Finally, the actuator’s performance may change over time, and this approach can be quickly repeated to update the model.

#### 2.2.2 The back-solver system model

In a physiotherapy setting, it is desirable to control the angular ranges and frequencies of joint motion. Further, smooth motion lends itself to patient safety. Toward this end, we created a Back-Solver model. While the dynamic model uses an input pressure curve to predict a leg trajectory, the Back-Solver uses dynamic equations to convert a desired leg trajectory into a requisite pressure input. This open-loop pressure input approach reduces the technical and equipment requirements for the platform in an end-user operated context, while ensuring safe and accurate leg motion.

First, the desired trajectory ( θ ) is numerically derived twice to find the angular velocity ( ω ) and angular acceleration ( α ) over time. For each time point, the net torque in the system ( $T_{net}$ _)_ is calculated using the angular acceleration (Eq. 1), the damping torque ( $T_{damp}$ ) using the angular acceleration (Eq. 2), and the torque due to the force of gravity ( $T_{gravity}$ ) using the leg angle (Eq. 3). The moment of inertia ( I ) is calculated by treating the leg as a point mass at the foot and one at the lower leg’s center of gravity, while the damping coefficient ( c ) is generated empirically by adjusting the dynamic model step response to match that of calibration step-input experiments. In Eq. 3, g is the acceleration due to gravity, $m_{f}$ and $m_{l}$ are the masses of the foot and lower leg respectively, $l_{ll}$ is the length of the lower leg and $f_{ll}$ is center of mass location for the lower leg as a percentage of segment length taken from the knee joint (∼43%, see Supplementary Material). The requisite actuator torque ( $T_{act}$ ) is then solved based on Eq. 4 and finally compared to a Torque-Theta profile (TTP), a mapping of the actuator’s FCP from a force-length to a torque-angle space (see Supplementary Information). The $T_{act}$ at each time point is converted into a pressure ( $P_{act}$ ) by dividing by the TTP value, based on the linear relationship between force output and pressure (Eq. 5).

$$T_{net}=I\;\alpha$$ (1)

$$T_{damp}=c\;\omega$$ (2)

$$T_{gravity}=-g\;\left(l_{ll}m_{f}+\left(l_{ll}\;f_{ll}\right)m_{l}\right)\;\cos\left(\theta \right)$$ (3)

$$T_{act}=T_{net}-\left(T_{damp}+T_{gravity}\right)$$ (4)

$$P_{act}\left(t\right)=\frac{T_{act}}{TTP\left(\theta \left(t\right)\right)}$$ (5)

Figure 2 depicts the workflow of this approach, combining the FCP from the quasi-static characterization process with a target angular trajectory to solve for a pressure input curve for the regulator.

### 2.3 Experimental methods

#### 2.3.1 Quasi-static testing using various clippings configurations

To characterize the BVA and evaluate the effect of the clipping mechanism on its output force and contraction, we performed a force-contraction experiment using a custom-made tensile test setup. An eight-cell BVA was built with a cross-section of 30 × 15 mm, with a distance of 15 mm between rings. The BVA was attached by thread to a load cell (SEN-14729, SparkFun Electronics) on one end and a pulley controlled by a stepper motor in the other end. To obtain the FCP, we loaded the actuator with a 5N pretension force using the stepper motor and load cell feedback, then applied a constant −20 kPa using a vacuum pressure regulator (IRV10A-C06LZN, SMC Pneumatics Inc.). The actuator was allowed to contract at a rate of 100 mm/min, controlled by the stepper motor, and the output force over time was recorded. The experiment was repeated in four configurations (zero, one, three, and five clipped cells) and three times for each configuration (*n* = 3).

#### 2.3.2 Proxy infant leg platform

**FIGURE 3:**
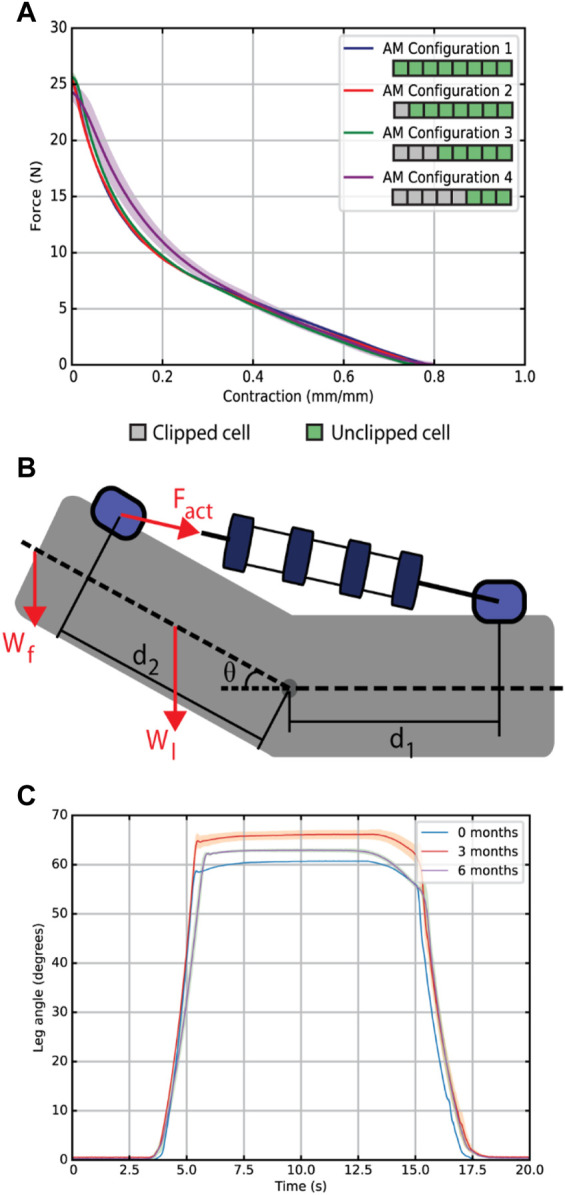
**(A)** A comparison of the force-contraction profiles for various clipping configurations at a constant pressure with a length-based scaling. **(B)** Schematic of the BVA on the infant leg model in the prone position indicating the actuator anchor positions $\left(d_{1}\right.$ and $d_{2})$ as well as the forces involved in knee flexion-extension (weight of the foot $W_{f}$ , weight of the leg $W_{l}$ and the actuator force $F_{act}$ ). The output leg angle is measured from an initially flexed position. **(C)** Results of the growth validation experiment. For 0, 3 and 6 months, the leg configuration and actuator anchor positions were adjusted to match the anthropomorphic data for infants of that age, and the corresponding actuator clipping was used. A step input of the same negative pressure was applied for the 0, 3 and 6 months configurations and the leg angle θ was measured.

A testing platform was designed as a proxy for a baby leg, as seen in Figure 3B and Supplementary Figure S4, so that the BVA could be tested in a variety of configurations corresponding to various infant ages [(Sun and Jensen, 1994; Wells et al., 2002), see Supplementary Table S2]. The leg proxy consists of two rigid sections connected by a pinned joint, representing the thigh and lower leg, as well as weights with adjustable positions to allow the mass and center of gravity of the leg to be changed depending on the simulated age of the infant (See Supplementary Table S3). Anchor points allow the actuator to be attached to the proxy leg, close to the hip and ankle locations. The anchors can be adjusted to allow different anchoring positions, different simulated leg lengths, and different infant legs. A prismatic joint was used to simplify the knee joint, despite its off-axis degrees of freedom, because flexion-extension was the focus of the study. Previous work has similarly simplified biological joints (Russo et al., 2021), though as mentioned in Section 2.1, future work will involve ensuring off-axis loads are safely constrained. Please refer to Supplementary Material for more details on the proxy leg design.

#### 2.3.3 Simulated leg growth experiment

To validate the actuator’s growing capacity for our specific case study, we tested the proxy leg with a sinusoidal pressure input curve. A single BVA performed the knee flexion-extension exercise in the infant model leg at 0, 3 and 6 months of age, defined in Supplementary Table S2. One cell was clipped for the 0-month configuration, while no cells were clipped for the 3 and 6 months scenarios. The setup was composed of a one stage vacuum pump (RS-2) that applies negative pressure to a vacuum chamber and an electronic vacuum pressure regulator (ITV0090-3MS, SMC Pneumatics Inc.). The pressure of the AM is set through the electronic regulator, and a vacuum pressure sensor (MPXV4115V) was connected in series to measure pressure near the BVA. In the infant leg model, the BVA is mounted through Kevlar thread as it is shown in Supplementary Figure S4. Finally, the input pressure curve is transmitted through a DAC (MCP4725) to the pressure regulator and the leg angle is recorded by video. The angle data was extracted in a video processing software (Tracker, Open Source Physics). For each month configuration, the knee flexion-extension exercise was repeated ten times (*n* = 10).

#### 2.3.4 Quasi-static actuator characterization for dynamic model

This section describes the experimental approach used to empirically derive the actuator’s FCP, to be used in the Dynamic Model described in Section 2.2.1. First, the actuator in the leg assembly was given a slow sinusoidal input pressure with a period of 60 s, allowing a full range of angular motion (in our case, 10–70deg, 0–9.5 kPa). The regulator output pressure and leg angle were measured over time, leg angle measured using an Inertial Measurement Unit (Adafruit BNO055). Secondly, the dynamic model used the angle data to solve for both the actuator length (based on geometry) and actuator force (based on required torque) over time. The force-time curve was then scaled by the input pressure curve using the linear scaling relationship between output force and actuator pressure (Gollob et al., 2021). Using these scaled force-time and contraction-time curves, the model can solve for the force-contraction curve for the actuator—multiple cycles of this quasi-static experiment were performed to create a consistent representation of the actuator FCP.

Hysteresis affected the trajectory. The final step is to fit a curve to this data to create a force-contraction curve that the dynamic model can reference. Because the model does not capture hysteresis and because the most significant target is the maximum angle reached, we fit the model FCP to the contraction section of the hysteresis curve, as this is the force that dictates the stopping point of the contraction before lowering the leg. The experimental FCP data with hysteresis and the fit FCP curve can be seen in Figure 2.

#### 2.3.5 Experimental testing for back-solver experimental validation

To validate the back-solver’s ability to produce an open-loop pressure input curve, the proxy leg model was set to patient parameters for a 3-month old infant, and a variety of desired sinusoidal knee angle trajectories were tested, with BVA pressure inputs solved from the back-solver. A pneumatic system with controllable pressure was set up, with a digital vacuum regulator (ITV 2091-21N2BS5, SMC Pneumatics Inc.) able to produce input pressure curves as commanded by an Arduino Mega, which also read the sensor outputs from a pressure sensor (MPXV4115V) and an orientation sensor (Adafruit BNO055). The regulator input was connected to a constant pressure source comprised on a manual pressure regulator (IRV10A-C06LZN, SMC Pneumatics Inc.) set to −50 kPa, which was connected to a vacuum chamber set to a lower pressure via a vacuum pump (RS-2, HBS).

## 3 Results

### 3.1 Quasi-static test using various clippings configurations

We conducted a quasi-static test to compare the force-contraction profile of the BVA using the clipping mechanism. Figure 3A shows a comparison of the BVA output force-contraction profile for varying clipped states, three trials each at an input pressure of −20 kPa. Contraction is calculated as the contracted distance over the full contractile length of the actuator. As we would expect from a VPAM, we observe a non-linear trend, with a larger output force earlier in the contraction. The maximum output force on the unclipped BVA was 25.6 ± 1.2 N. A non-significant difference appeared in the maximum output force of the unclipped VPAM and each of the clipped configurations. Discrepancies between an unclipped VPAM and a clipped VPAM are likely due to slight differences in the arrangement of the rings in the fabrication, and the crumpled and compressed skin in the clipped cell (see Supplementary Material). Most importantly, various clipping configurations didn’t affect the output FCP of the BVA.

### 3.2 Simulated leg growth experiment

Figure 3C shows the results of our simulated leg growth experiment, where the leg proxy geometry and masses were adjusted to simulate infant growth. The model represents an infant’s leg, from below the hip, in the prone (face-down) configuration used by physicians for passive knee ROM exercises (Passo, 1974).

We defined a benchmark value of 54° for our BVA when actuated in the infant leg model, based on the ROM of the knee for a kicking motion of healthy infants at 3 months of age (Sargent et al., 2015). The BVA produced a knee angle of 60.66° ± 0.6° at 0 months of age, 65.40° ± 0.6° at 3 months of age, and 62.54° ± 0.6° at 6 months of age. Experimental measurements on the infant leg model surpassed our target range of motion by ∼13%. Differences between experimental results in each month are likely due to slight discrepancies in BVA assembly (e.g., ring misalignment) and sealing process issues (see Supplementary Material). The membrane can affect the output contractile length as it can become folded between two rings in pattern that allows easier contraction. The output angle in the experimental results is proportional to contraction of the BVA. Therefore, as expected, when one cell was clipped the maximum knee angle decreased due to the reduction in the total contractile length of the BVA. Our experimental values of ROM for therapy exercises fall within reported values (Mendoza et al., 2021).

In addition to the angular range of flexion, the speed of the ROM exercise is relevant. The peak angular velocity obtained from our experiments was greater than $55\;{}^{\circ}.s^{-1}$ for knee flexion. These results indicate that the BVA can produce the knee flexion-extension exercises in less than the required time of 3 s (according to clinical collaborators), and the operation window scan be tuned to ensure the patient’s safety.

### 3.3 Back-solver experimental validation

**FIGURE 4:**
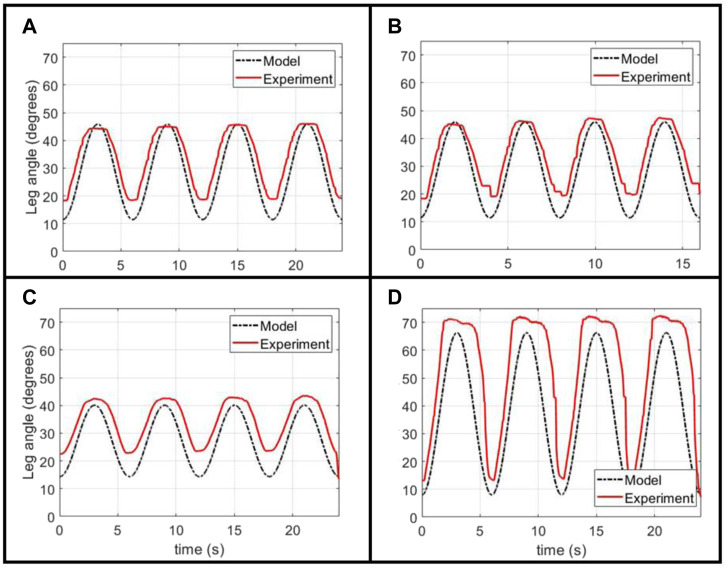
Experimental validation of the Back-Solver model applied to an unclipped actuator, given a variety of target sinusoidal angular trajectories: **(A)** a baseline trajectory with a 6 s period, **(B)** a 4 s period trajectory of the same magnitude, and **(C*,* D)** two 6 s period trajectories with different target angle values.

To assess the performance of our model as an approach to open-loop control of the leg, we derived pressure inputs curves for different sinusoidal target trajectories and compared how closely the experimental trajectory matched the target, as seen in Figure 4.

This open-loop input can closely follow the shape and angular magnitudes for different sinusoidal targets, ensuring a smooth flexion and extension motion that is a key safety design parameter for our physiotherapy platform. The experiment tends to over-predict the minimum angle of the trajectory, a result that can be explained well by the actuator’s hysteresis mentioned before. Given that our model does not capture the increase in actuator force output after contraction, it over-estimates the required pressure in the lowering of the leg. We believe that the result of this over-estimation is the experimental minimum angle being above the target minimum. Another possible source of discrepancy between the target and experimental trajectory is the divergence of the pressure regulator from following the prescribed target pressure curve due to the regulator’s internal control scheme, as was observed in experimental measurements (Supplementary Figure S8).

Finally, we demonstrated the ability of the same model to predict pressure inputs when the actuator is clipped. We use the same FCP derived from the quasi-static unclipped experiment, while normalizing the *x*-axis by the new length of the actuator, as the scaled FCP for a bellows actuator is not affected by the number of cells (as seen in the results from Figure 3A). With this scaled FCP, the same process is used as for the unclipped modelling.

**FIGURE 5:**
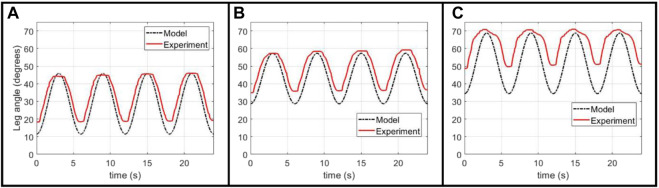
Experimental validation of Back-Solver model applied to a clipped actuator, comparing the performance between **(A)** an unclipped actuator and **(B*,* C)** two different target trajectories for an actuator with two clipped cells.

Figure 5 shows the performance of the model-generated pressure curve, calculated for an unclipped actuator and an actuator with two clipped cells out of eight. As with the unclipped case, the open-loop curve can closely approximate the target shape and maximum angles, thought the lower-angle is over-shot. This effect is exacerbated when the minimum angle value is increased, and we hypothesize this is due to the greater discrepancy in the hysteretic profile in the middle range of the actuator’s contraction (as opposed to the extremes of full contraction and extension), as is achieved when a larger lower-angle is used.

Through this, we show that the Back-Solver model is able to generate an open-loop curve for the system with angular magnitudes and shapes similar to the desired target trajectory, but that there are some discrepancies, particularly in the over-shooting of the minimum angle, most probably due to actuator hysteresis. For a closer matching between target and experimental trajectories, further work is needed to model the hysteretic behavior of the actuator and develop a closed-loop nonlinear controller that can produce a more controlled pressure output.

## 4 Discussion

In this paper we presented a concept for a BVA with a modular layout that enables the adjustment of the actuator’s contractile length through the clipping and releasing of cells, so that it can adapt to a variable working space and target range of motion. The FCP of the actuator was characterized at various clipped states, demonstrating that the clipping of cells does not affect the shape of the output FCP, while allowing for a tuned contractile length. A case study was presented, inspired by physical therapy in infants with myelomeningocele, who require regular range-of-motion exercises of their paralyzed lower limbs during their early development. We simulated the growing infant leg through a leg proxy setup, and demonstrated the applicability of the BVA as a wearable actuator for growing patients, with one actuator adapting to changing contraction lengths and output force requirements over 6 months of a patient’s development. We present a dynamic model that generates model-based open-loop pressure control curves to move the system in a desired angle-time trajectory, with good agreement in terms of shape and target angles. In our case study, this control would mean successful exercise range of motion and smooth motions for the patient, but further demonstrates the generalizable predictability of this actuator for modelling in other applications. We demonstrated that our BVA can perform successfully in our specific case study in terms of range of motion and cycle time for the therapy exercise, across different months.

There are some weaknesses in our current BVA design and systems approach. On the actuator side, the skin material is vulnerable to failure over multiple cycles, leading to an insufficient life cycle. The clipping mechanism is currently a manual procedure, which, though sufficient for a medical device application such as this, could be made autonomous in the future for greater applications in robotics and autonomous systems. For more accurate control of leg motion, it is important to develop and incorporate closed-loop control of the system, which might include a model-based control approach that can better overcome the nonlinearities in the force output of the BVA. A closed-loop control would also be essential for patient safety in a wearable application.

With improvements, the application space for our length-adjustable BVA is vast. Looking beyond physical therapy applications, the BVA can be used as a prismatic joint in graspers of manipulators, as well as exploration robots that could adapt to shifting workspace or actuation requirements. The clipping or deactivating of sections could repair leaks in damaged actuators, or even adapt actuator geometry based on repeated usage requirements analogous to how biological muscles remodel with repeated use.

## Data Availability

The raw data supporting the conclusion of this article will be made available by the authors, without undue reservation.
