# Supplementary material for: A length-adjustable vacuum-powered artificial muscle for wearable physiotherapy assistance in infants
Source: Front Robot AI. 2023 May 4;10:1190387. doi: 10.3389/frobt.2023.1190387 (PMC10192875; doi:10.3389/frobt.2023.1190387)
Supplement: Supplementary file 1 [file DataSheet1.PDF]

Supplementary Material

A Length-Adjustable Vacuum-Powered Artificial Muscle for Wearable Physiotherapy Assistance in Infants

Samuel Dutra Gollob[1]†, Mijaíl Jaén Mendoza[2*]†, Bon Ho Brandon Koo[1], Esteban Centeno[2], Emir A. Vela[2,3], Ellen T. Roche[1,4]

*** Correspondence:** Ellen T. Roche: etr@mit.edu

†These authors contributed equally to this work and share first authorship

1 Supplementary Data

1.1 Fabrication of the artificial muscle with different number of cells

To fabricate the VPAMs, we first prepared the materials and manufactured the required components. **Figure S1** shows all key elements for assembling a single artificial muscle, and a complete list of all required elements is outlined in **Table S1**.

The preparation of the membrane material (Polyethylene film, Policlick) consisted of cutting a rectangular piece of polyethylene PE sheet (thickness: 0.100 mm). This film was marked with a designed space for the rings to guide the assembly of the artificial muscle, and it was then thermally sealed using a heat sealer (HD-300M-IR, Hurricane) as a sleeve to slide the internal rings within it as shown in **Figure S2**. Additionally, the excessive film is removed near the flat band produced by the heat sealing.

Each part of the AM (internal, ending, and external rings) was 3D-printed using polylactic acid (PLA, eSUN) in a 3D-printer Prusa MK3s. The internal and ending rings were designed to have a groove on its circumferential face, and the external rings were designed to fit into it. The internal rings were placed inside the membrane, and then these were fixed to the membrane by press-fitting the external rings into membrane-internal ring groove. To fixate each internal ring, two external rings are required and bonded together on the corners using cyanoacrylate adhesive (Triz, Industrial Beta). To ensure an airtight sealing, epoxy syringe glue (5-minute Epoxy, Adhaero) is adhered to the groove of the ending parts of the actuator before its location in the membrane.

To adapt the contractile length of our artificial muscle, we built different set of anchoring parts to deactivate different number of cells, those were 3D-printed using thermoplastic polyurethane (TPU, eSUN) for easing the deactivation process. One artificial muscle of 6 cells is shown in **Figure S3**, with the following characteristics. Cross-sectional area: 30x15, Distance between rings: 15 mm, and ring thickness: 6 mm.

1.2 Fabrication of the infant leg model

For experimental testing and evaluating the performance of the actuator for the rehabilitation exercise, we designed a robust and configurable leg model prototype capable of changing limb weights as well as its anchor points. To achieve this feature, the infant leg model uses interchangeable parts that are located in determined positions to replicate anthropometric characteristics of the baby as he grows up. Those characteristics are the circumferences, lengths and weights of the babies shown in **Table S2** (Sun and Jensen, 1994; Wells *et al.*, 2002). Currently, the infant model leg is useful to validate the artificial muscle at 0, 3 and 6 months of age for our experimental testing.

The infant leg model consists of different interchangeable parts indicated in **Table S3.** Two main parts that represent the thigh and the lower leg of an infant (part for thigh and lower leg length). Those are designed as square beams with pattern of holes that enable them to attach the interchangeable piece required for each month. For the knee flexion-extension motion, the leg model is designed with a revolute joint to have a unique degree of freedom in the sagittal plane. Fabrication process of the leg model mainly consisted of 3D printing the main parts and the interchangeable pieces using PLA and assembling all the components. The interchangeable parts for lower limb radiuses (part for thigh and lower leg radius) are used as anchor points for mounting the artificial muscle, and an inextensible cable (Kevlar) is added in those parts to transmit the actuator force to the leg model. In addition to that, the weights of the lower leg and the foot are considered as points load using one foot and two lower leg mass containers for metal disc masses. Those are added in the containers to achieve the weight desired for each month.

To validate the artificial muscle for our case study, the infant leg model is implemented for our experimental setup in the flexion-extension exercise of the infant therapy as shown in **Figure S4**. The infant leg model has a fixed part during the exercise, and an initial angle support to start each cycle of knee flexion-extension in the same angle.

1.3 Functional requirements and considerations for infants affected by myelomeningocele

Understanding the medical issue in physiotherapy for infants with myelomeningocele (MMC) was the first step for the design process in our specific case study. We identified the functional requirements and considerations through discussions with clinical collaborators, and clinical guides, as well as literature about myelomeningocele. All the required characteristics for physical therapy in infants with MMC for our VPAM are summarized in **Table S4**.

The leg movement of infants with MMC is limited and reduced during their growth, and in our space limited scenario produced by the infant anthropometric measurements, we considered to develop a light weight and low profile artificial muscle to avoid interfering with infant voluntary movements. From the physical therapy for lower limb rehabilitation of the babies, one of the most important exercises is the knee flexion-extension exercise (Seattle Children’s, 2018) due to it is a primary movement involved in the development of complex motions such as crawling and walking. Thus this exercise was chosen for our specific case study. The main output of the exercise is the knee angle produced. To validate the performance of the artificial muscle in the exercise, a benchmark value was defined for our actuator to reach when actuated to an infant leg model in each month. The value selected was 54 degrees as it is the range of motion (ROM) of the knee angle for a kicking motion for heathy infants at 3 months of age (Sargent *et al.*, 2015). In addition to the desired ROM, the newborn (infant leg model) for our validation is laid in a horizontal surface in prone position reproducing the position

of the baby in the physical therapy (Passo, 1974). Our period of treatment, time for leg flexion and anchor supports for our AM were set based on suggestions from our clinical collaborators to provide an early treatment from a few weeks of birth to 6 months of age.

1.4 Artificial muscle selection for infant therapy

To design an artificial muscle for our specific case study, first we developed a quasi-static model to identify the force-contraction FC profile of the AM during our knee flexion-extension exercise. **Figure S5** shows a simplified diagram of the forces and the geometrical dimensions involved throughout the exercise. To generate knee flexion motion, the torque produced by the actuator force ($\boldsymbol{T_{act}}$) should be equal or exceed the counter torque caused by the weight of the foot ($\boldsymbol{W_f}$) and the weight of the lower leg ($\boldsymbol{W_l}$).

$$\boldsymbol{T_{act} = W_f l_{ll} \cos(\theta) + W_l l_{ll} f_{ll} \cos(\theta)} \tag{1}$$

Where $\boldsymbol{l_{ll}}$ stands for the length of the lower leg and $\boldsymbol{f_{ll}}$ stands for center of mass location for the lower leg as a percentage of segment length taken from the proximal joint (~43%).

To calculate the actuator force, the length of the actuation system ($\boldsymbol{L_{act}}$), and the lengths between each anchor point and the knee ($\boldsymbol{u}$, $\boldsymbol{v}$) were calculated:

$$\beta = \pi - \theta - \tan^{-1}(\frac{r_t}{d_1}) - \tan^{-1}(\frac{r_{ll}}{d_2}) \tag{2}$$

$$v = \sqrt{{r_t}^2 + {d_1}^2} \tag{3}$$

$$u = \sqrt{{r_{ll}}^2 + {d_2}^2} \tag{4}$$

$$L_{act} = \sqrt{u^2 + v^2 - 2uv\cos(\beta)} \tag{5}$$

Where $\boldsymbol{\beta}$ is the angle between the lines $\boldsymbol{u}$ and $\boldsymbol{v}$, $\boldsymbol{r_t}$ is the radius of the thigh, $\boldsymbol{r_{ll}}$ is the radius of the lower leg, and $\boldsymbol{d_1}$and $\boldsymbol{d_2}$ are the horizontal distances from the knee to the anchor points on the thigh and the lower leg, respectively. Those distances are based on the anthropometric measurements shown in **Table S2**, and through this process, the model enabled us to calculate the absolute length of the actuation system $\boldsymbol{L_{act}}$, in other words, the space available for the artificial muscle.

Using the quasi-static model, the required force of the actuator $\boldsymbol{F_{req}}$ and the required length contraction $\boldsymbol{s_{req}}$ can be estimated given the range of motion of $\boldsymbol{\theta}$ from **Table S4**.

$$\gamma = sin^{-1}(\frac{v.\sin(\beta)}{L_{act}}) \tag{6}$$

$$F_{req} = \frac{(W_l\, l_{ll}\, f_{ll}\, \cos(\theta) + W_f\, l_{ll}\, \cos(\theta))}{u\sin(\gamma)} \tag{7}$$

$$s_{req} = L_{act}(\theta_0) - L_{act}(\theta) \tag{8}$$

Where γ is the angle between L_{act} and u.

From the analysis, suitable anchor points were identified as close to the hip and the ankle. To ensure safety of the user, we chose those points, because the maximum force required is less than in other scenarios. However, there is trade-off between the contraction required and the force required, which means that our FC profiles selected for each month have high required contraction (>80 mm).

As shown in **Figure S4**, the L_{act} is composed by two physical components, the artificial muscle and an inextensible cable. Those components are represented by the absolute length of the artificial muscle, l_{am}, and the length of the inextensible cable, l_{ic}.

$$L_{act} = l_{am} + l_{ic} \tag{9}$$

The absolute length of the artificial muscle is represented by non-rigid elements (flexible membrane) and rigid elements (rings). Thus, we defined two lengths associated to each element, the contractile length l_c and the fixed length l_f of the artificial muscle considering the locking mechanism, as can be seen in equations below:

$$l_{am} = l_f + l_c \tag{10}$$

$$n_{total} = n_{act} + n_{deact} \tag{11}$$

$$l_f = t_r\,(n_{total} + 1) \tag{12}$$

$$R = \frac{B}{D} \tag{13}$$

$$l_c = R * n_{act} \tag{14}$$

One single artificial muscle has a defined number of cells n_{total}, and a number of deactivated cells n_{deact}, as well as activated cells n_{act} to operate. The fixed length l_f and contractile length l_c are calculated in equation 12, 13 and 14, where t_r stands for thickness ring of the artificial muscle, R stands for the AM aspect ratio, B stands for the distance between rings and D stands for the height of the artificial muscle cross-sectional area.

Based on the defined actuation system length during the exercise for each month, we then dimensioned the geometrical parameters of our artificial muscle. We analyzed one actuator with an aspect ratio R = 1.0 and a cross-sectional area of 30x15 mm to estimate the number of cells required and the contraction required for each month. For our AM, the suitable contraction ratio was set as 90 % in each month due to difficulty to achieve a fully contracted state as the contraction ratio of our AM is limited by the thickness of the membrane when it is compressed between rings during actuation. Further, the suitable ring thickness was set as 6 mm due to its viability and easy fabrication through 3D printing. Varying the ring thickness, we found that t_r = 6 mm is a suitable physical dimension for the AM, and identified that the AM should have 7 activated cells for the first three months of age, then it should use 8 activated

cells until of the treatment as shown in **Table S5**. We built an artificial muscle with a total of 8 cells for the validation of the knee flexion-extension exercise during growth.

1.5 Effect of clipping in different locations

To explore the effect of clipping different cells, we built a VPAM with 8 cells using 0.1 mm PE film. Its cross-sectional area was 30x15 mm and R = 1. We conducted force contractions experiments varying a 2-cell locking in different locations at constant pressure of -20 kPa. The experiment was repeated three times for each location (n=3). In the different examples shown in **Figure S6**, the locking mechanism was able to deactivate any pair of cells of the VPAM, and produce a similar maximum output force falling within the ranges of values of 35.6 ± 0.6 N. Our experiment demonstrates that the effect of clipping in different locations have an insignificant effect in terms of output force and contraction.

1.6 Dynamic Model of Actuator

Before using the back-solving dynamic model to predict the pressure inputs for a target leg trajectory, the dynamic model was validated via dynamic motion experiments with the leg, where the pressure from the regulator was used as the input to a dynamic model in Simulink (Mathworks). As can be seen in Figure S7, the model follows the experimental results closely, particularly in flexion. During extension, there is some divergence, most probably due to the hysteresis observed in the actuator. The model's lack of hysteresis effect means it under-estimates the actuator's force during extension – in other words, the model actuator provides less resistance to the leg's descent than the experimental case.

Further, a key detail of the Back-Solving Model translates an actuator force into a torque value applied around the knee joint of the leg. Using the same element naming terminology used in Figure 3b and S5, the pulling force applied by the actuator on the leg ($\boldsymbol{F_{act}}$) generates a torque ($\boldsymbol{T_{act}}$) calculated as:

$$\boldsymbol{T_{act}} = \boldsymbol{u} * \boldsymbol{v} * \frac{\boldsymbol{F_{act}} * \mathbf{sin}(\boldsymbol{\beta})}{\boldsymbol{L_{act}}} \tag{15}$$

Where β is the angle between the lines u and v.

To convert the FCP of the actuator to its TTP, the contraction of the actuator (s) at a given leg angle (θ) is found by subtracting the L_{act} value for that angle from the L_{act} for the initial starting angle (θ_0). The FCP value at that contraction is then converted to the torque value for θ.

$$s(\theta) = L_{act}(\theta_0) - L_{act}(\theta) \tag{16}$$

$$TPP(\theta) = u * v * \frac{FCP(s(\theta)) * \sin(\beta)}{L_{act}(\theta)} \tag{17}$$

1.7 Pressure regulator performance

Figure S8 shows how the chosen pressure regulator does not perfectly follow the target curve, as the regulator is driven by its own internal controller and in our experiments operates in the lower part of its operating range.

2 Supplementary Figures and Tables

2.1 Supplementary Figures

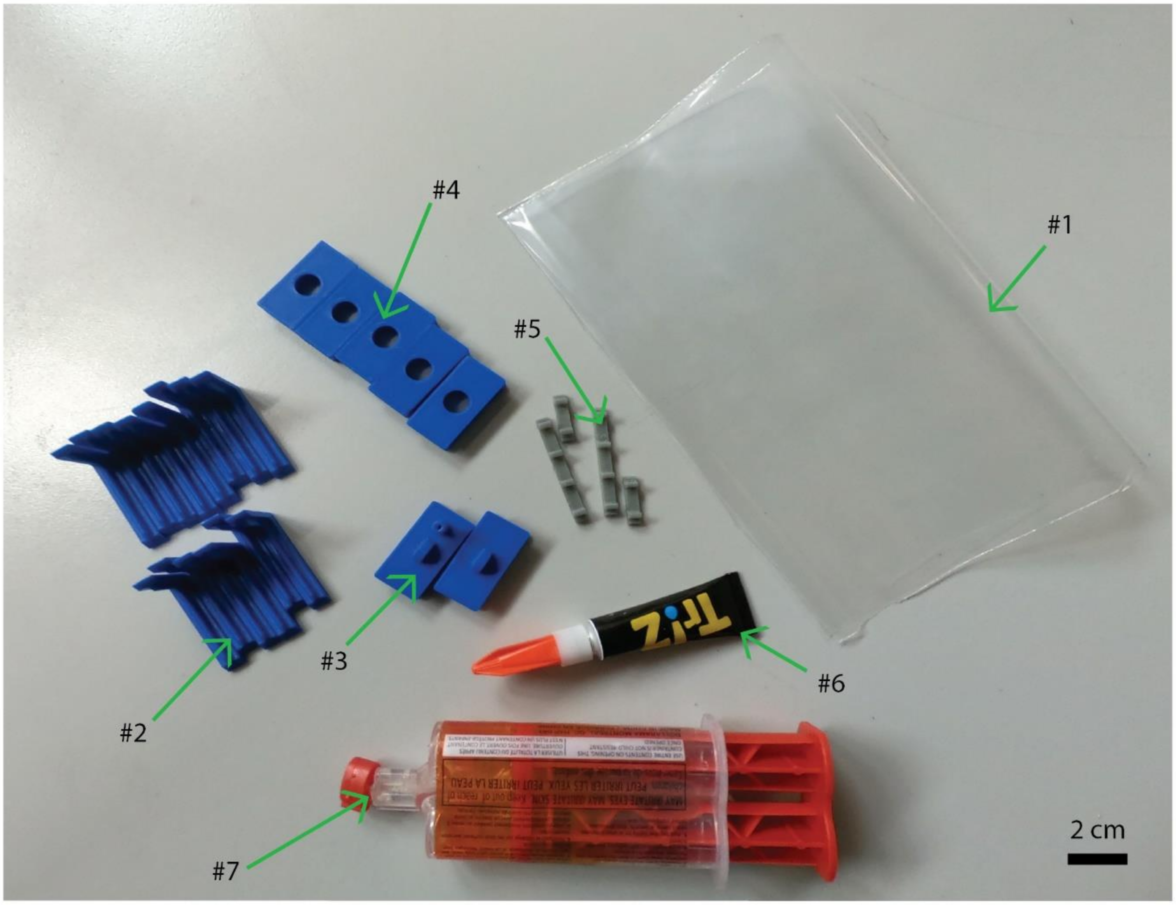

Supplementary Figure 1. Materials and components for constructing an artificial muscle

Supplementary Table 1. Materials and components list for an artificial muscle

Number	Component
#1	PE film
#2	external rings
#3	ending rings
#4	Internal rings
#5	Anchoring parts

#6	Cyanoacrylate adhesive
#7	Epoxy syringe glue

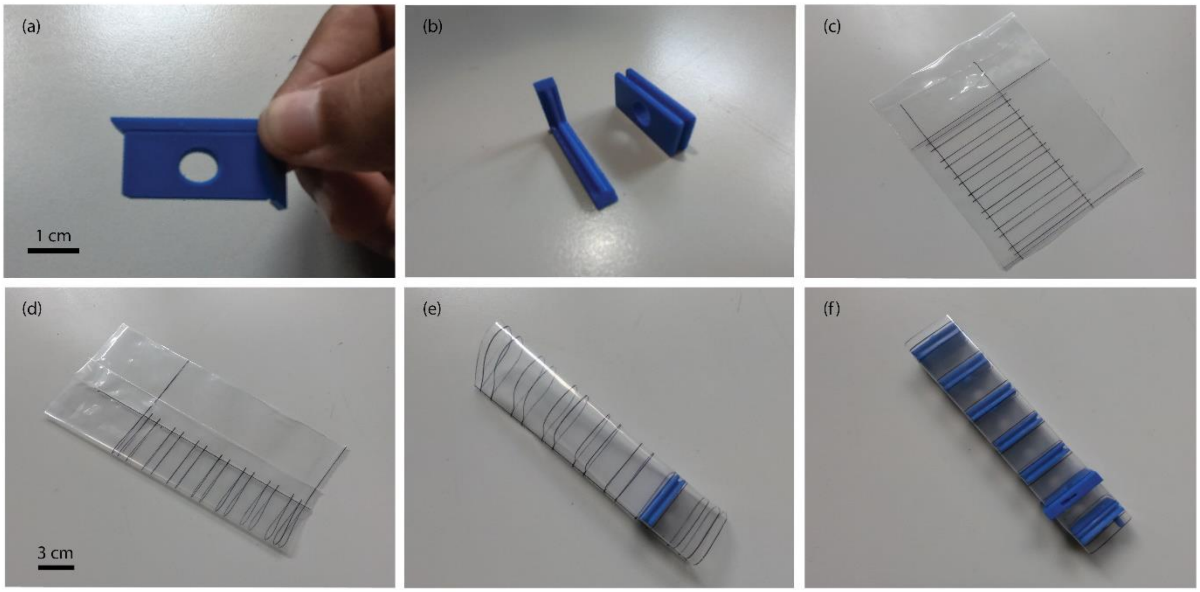

Supplementary Figure S2. Assembling the rings to the film with a designed space

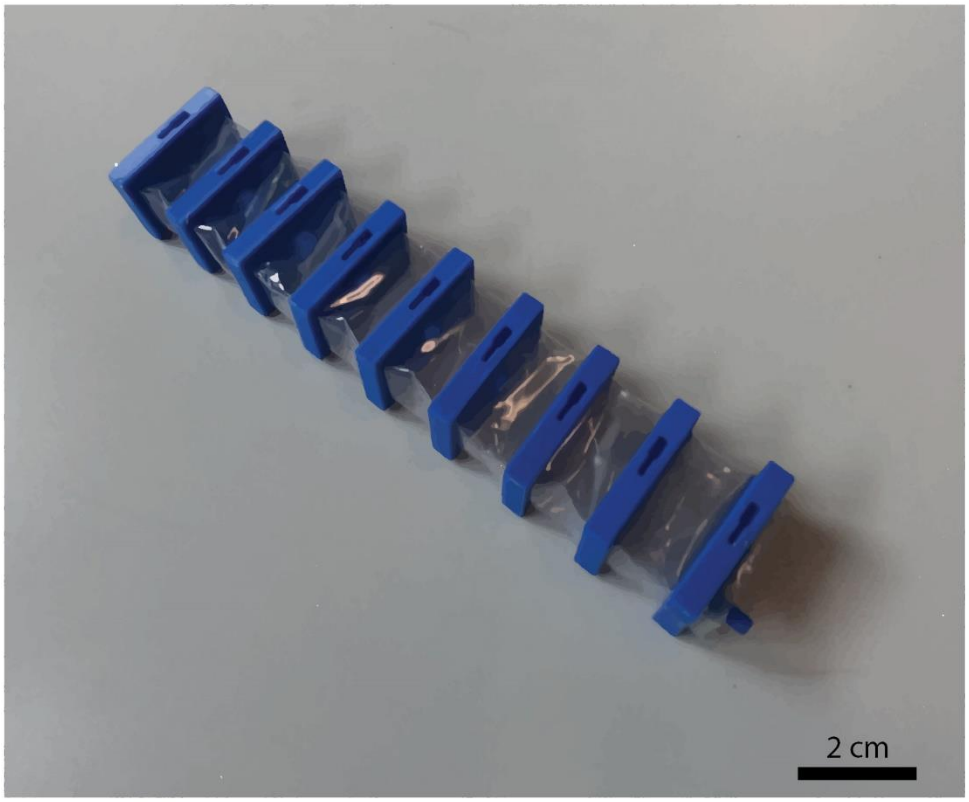

Supplementary Figure S3. Fabricated VPAM with 8 cells.

Supplementary Table S2. Anthropometric measurements and weights of lower limb of infants, based on (Sun and Jensen, 1994; Wells *et al.*, 2002)

Age (months)	Foot mass (g)	Lowe leg mass (g)	Thigh mass (g)	Lower leg length (mm)	Thigh length (mm)	Lower leg circumference (mm)	Thigh circumference (mm)
0	52	161	295	94	94	22	32
3	78	217	415	103	106	23	35
6	103	260	537	112	118	25	38

Supplementary Table S3. Components list for the infant leg model for experimental setup in AM validation

Number	Component
#1	Fixed part
#2	Part for thigh radius

#3	Part for lower leg radius
#4	Part for thigh length
#5	Part for lower leg length
#6	Lower leg mass container
#7	Foot mass container
#8	Initial angle support
#9	Artificial muscle
#10	Kevlar cable

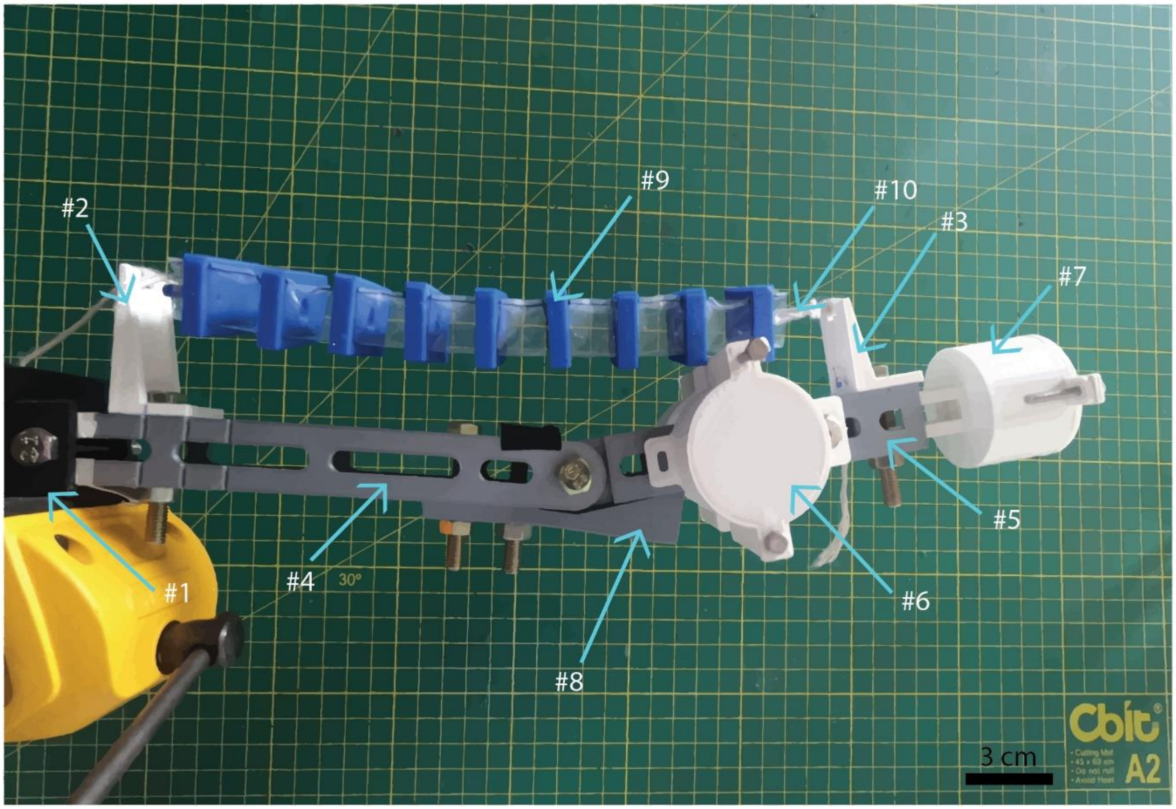

Supplementary Figure S4. Infant leg model for experimental setup for knee flexion-extension exercise in prone position

Supplementary Table S4. Design parameters for the artificial muscle in rehabilitation therapy for infants with myelomeningocele

Design Considerations	Characteristics
Light weight	≤ 100 g
Low profile	≤ 20 mm

Rehabilitation exercise	Knee Flexion-Extension
Minimum range of motion	20 ° - 74 ° (0 ° = fully extended, 90 ° = right angle at knee)
Newborn position [a)]	Prone
Baby age [a)]	From birth to 6 months
Time for leg flexion [a)]	3s flexion, 3s relaxation
Anchor supports [a)]	Thigh or hip, and ankle

[a)] Recommendations from our clinical collaborators at Instituto Nacional de Salud del Niño de San Borja

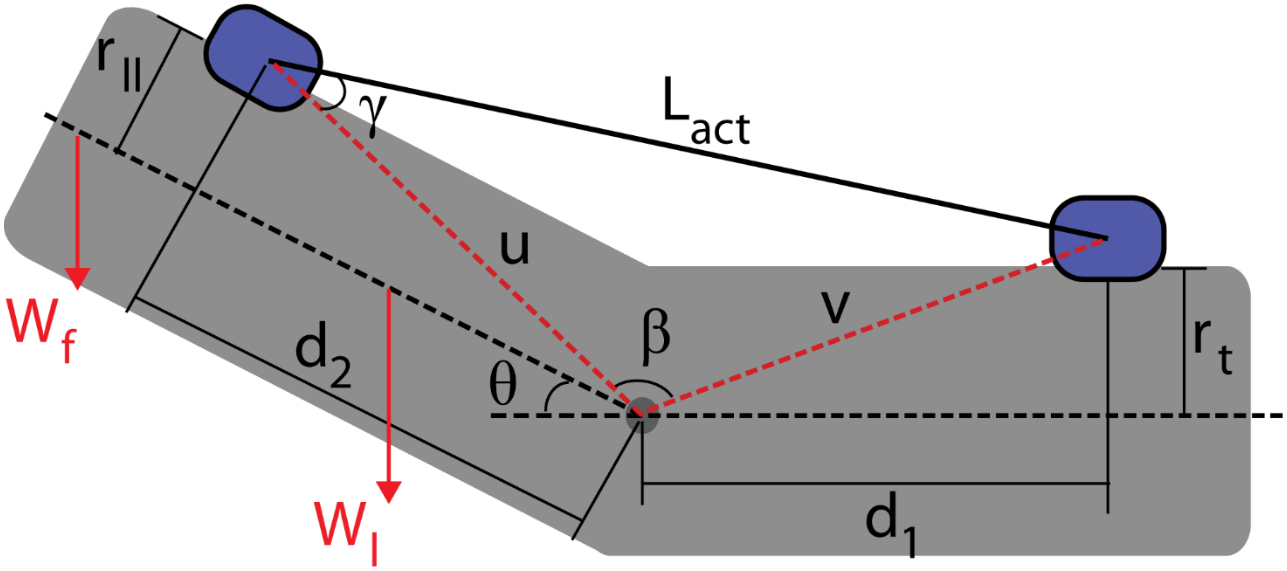

Supplementary Figure S5. Simplified diagram showing the length produced during the knee flexion-extension in prone position

Supplementary Table S5. Geometrical parameters for the artificial muscle in rehabilitation therapy for infants with myelomeningocele

Age (month)	Actuation system length (mm)	Contractile length of the AM (mm)	Fixed length of the AM (mm)	Inextensible cable length (mm)	Number of activated cells	Absolute Contraction (mm)	Contraction
0	172.59	105	54	13.59	7	91.10	87 %
1	179.10	105	54	20.10	7	93.82	89 %
2	185.76	105	54	26.76	7	96.61	92 %
3	192.43	120	54	18.43	8	99.39	83 %

4	199.10	120	54	25.10	8	102.17	85 %
5	205.76	120	54	31.76	8	104.95	87 %
6	212.43	120	54	38.43	8	107.72	90 %

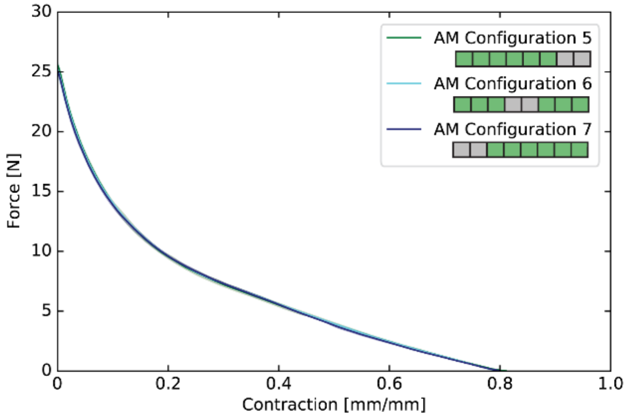

Supplementary Figure S6. A comparison of force-contraction profiles for varying clipped cell locations, demonstrating the clipping location does not affect the force profile. A green cell indicates an unclipped cell and a gray cell indicates a clipped cell.

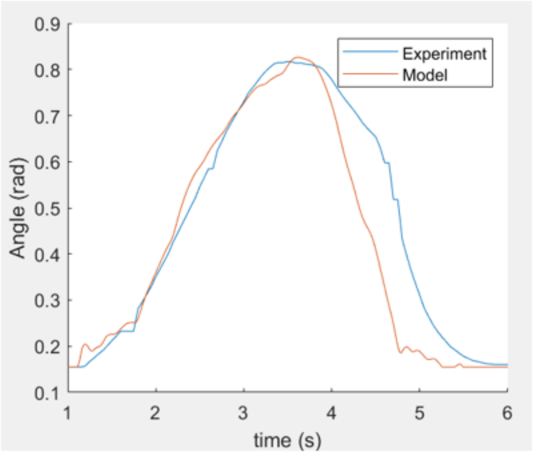

Supplementary Figure S7. Agreement between the model and experiment for a cycle of the leg trajectory over time, using the pressure input from the experiment in the model.

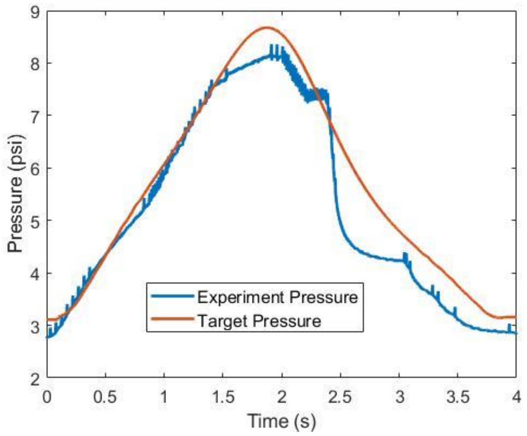

Supplementary Figure S8. Shows the output vacuum pressure magnitude for our vacuum regulator, given a target pressure signal, demonstrating how the regulator deviates from the target in an asymmetric manner.
